# Broad-spectrum antiviral brincidofovir inhibits Epstein-Barr virus and related gammaherpesvirus in human and nonhuman primate cells

**DOI:** 10.1172/JCI195764

**Published:** 2025-11-25

**Authors:** Abaigeal Donaldson, Madeleine R. Druker, Maria Chiara Monaco, Emily H. Stack, Paige Zimmerman, Amanda Lee, Izabela Bialuk, William Frazier, Irene Cortese, Heather Narver, Masatoshi Hazama, Fuminori Yoshida, Xiaofan Li, Laurie T. Krug, Stacey L. Piotrowski, Steven Jacobson

**Affiliations:** 1 Viral Immunology Section, National Institute of Neurological Disorders and Stroke (NINDS) and; 2 Translational Neuroradiology Section, NINDS, NIH, Bethesda, Maryland, USA.; 3 Department of General and Experimental Pathology, Medical University of Białystok, Białystok, Poland.; 4 Experimental Immunotherapeutics Unit and; 5 Animal Health and Care Section, NINDS, NIH, Bethesda, Maryland, USA.; 6 SymBio Pharmaceuticals Limited, Tokyo, Japan.; 7 HIV and AIDS Malignancy Branch, National Cancer Institute, NIH, Bethesda, Maryland, USA.

**Keywords:** Neuroscience, Virology, Multiple sclerosis

## Abstract

Epstein-Barr virus (EBV) is of growing interest for its potential role in neurodegenerative diseases such as multiple sclerosis (MS) and its possible utility as a therapeutic target in herpesvirus-associated chronic diseases. The effects of brincidofovir (BCV) on EBV reactivation were evaluated in vitro using EBV-infected spontaneous lymphoblastoid cell lines (SLCLs) and peripheral blood mononuclear cells (PBMCs) derived from patients with MS and healthy controls. In addition, a B lymphoblastoid cell line and PBMCs from common marmosets (*Callithrix jacchus*) naturally infected with an EBV-related gammaherpesvirus (Callitrichine herpesvirus 3, CalHV-3) were used to measure BCV efficacy in a nonhuman primate model. BCV significantly inhibited gammaherpesvirus reactivation, with decreased lytic and latent viral transcript expression. These results suggest that BCV may be a useful antiviral for inhibiting EBV activity in patients with MS. Additionally, this work further validates the utility of CalHV-3 in marmosets as a translational model for the investigation of successful EBV-targeting therapeutics.

## Abstract 2

Brincidofovir (BCV), a broad-spectrum antiviral agent, effectively reduces gammaherpesviruses in human and nonhuman primate cells.

## Introduction

Multiple sclerosis (MS) is a chronic progressive neuroinflammatory disease and the leading cause of nontraumatic disability in young adults (1). While the etiology of MS remains unknown, it is believed to involve the convergence of various genetic, environmental, and immunological factors (1). In addition to smoking, vitamin D deficiency, high latitude, and female sex, viral infection has long been implicated as a potential risk factor for the development of MS (2). While dozens of viruses have been proposed over the decades, evidence continues to grow for the role of Epstein-Barr virus (EBV) in MS (1, 3–5).

EBV is a ubiquitous gammaherpesvirus that infects over 90% of the global adult population (6). It is the leading cause of infectious mononucleosis (IM) and is most commonly acquired during childhood and early adolescence (7). After resolution of acute infection, EBV typically establishes subclinical lifelong latency in memory B lymphocytes (8). In a subset of individuals, however, chronic infection is associated with several malignancies including Burkitt lymphoma, nasopharyngeal carcinoma, gastric carcinoma, and Hodgkin lymphoma, as well as autoimmune diseases including rheumatoid arthritis, Sjögren’s syndrome, systemic lupus erythematosus, and, more recently, MS (9–12).

The hypothesis that EBV may contribute to the development of MS is supported by a growing body of epidemiological, serological, and virological evidence (1, 3–5, 13). In a recent landmark study that retrospectively evaluated the association between viral infection and MS in 10 million United States military personnel, it was reported that infection with EBV was associated with a 32-fold increased risk for developing MS (3). Additionally, the study followed 35 individuals who were EBV seronegative at baseline and developed MS at follow-up and reported that all but one (97%) seroconverted before MS onset, which contrasts with the seroconversion rate of EBV-negative individuals who did not develop MS (57%). Lastly, in the baseline-negative individuals who developed MS at follow up, serum concentrations of neurofilament light chain (sNfL), which is an early pathological change in MS indicative of neuroaxonal damage, increased only after EBV infection. Taken together, this study presented evidence suggesting that EBV infection is a common prerequisite for the development of MS (3). Similarly, others have reported that a history of IM is associated with increased risk for MS, that patients with MS have higher antibody titers to EBV antigens than healthy controls (HCs), and that patients with MS show an increased antibody response associated with EBV peptide tiles in serum and CSF samples following VirScan analysis using a virus discovery pipeline (4, 14–17).

The mechanism by which EBV may contribute to MS pathogenesis is believed to involve dysregulation of the host immune system, which culminates in CNS inflammation and demyelination (18). Animal models have been important for the better understanding of MS pathogenesis, including the role of herpesviruses in disease manifestation (19, 20). In particular, the common marmoset (*Callithrix jacchus*), a New World nonhuman primate, has provided a vital tool for the investigation of MS, showing that infection with human herpesvirus 6 (HHV-6) can accelerate the progression of an MS-like disease experimental autoimmune encephalomyelitis (EAE) (21, 22). The marmoset can be naturally infected with Callitrichine herpesvirus 3 (CalHV-3), a gammaherpesvirus that is phylogenetically related to EBV and shares many biological properties, including B cell tropism and association with B cell lymphoma (23–26). CalHV-3 has been reported to play a role in EAE manifestation in marmosets (27). The utilization of animal models, such as CalHV-3 infection in the marmoset, can aid the investigation of EBV-targeted antivirals and therapeutics, including those that may benefit patients with MS (28).

While great progress has been made in the ability to control and slow MS progression, in large part due to the advent of B cell depleting therapies, there remains no cure for this disease (29). This may be, in part, a reflection of current treatments aiming to modulate host immune responses rather than target underlying drivers of disease pathogenesis (30). Specifically, very little is known about the potential efficacy or benefit of modulating viral activity in patients with MS.

Brincidofovir (BCV) is a broad-spectrum antiviral agent and lipid-conjugate of cidofovir that has shown activity against a broad spectrum of double-stranded deoxyribonucleic acid (dsDNA) viruses by acting as an alternative substrate of DNA polymerase to inhibit viral replication (31). The intracellular active form of BCV is known as CDV-PP (cidofovir diphosphate), which structurally resembles dCTP as a natural substrate of viral DNA polymerase and can be incorporated into viral genome DNA instead of dCTP by viral DNA polymerase, leading to the inhibition of viral DNA replication (32). BALF5 is a subunit of the viral DNA polymerase and is thought to be the target of many anti-EBV therapies (33, 34). Although no direct evidence for CDV-PP activity against BALF5 is available, observations and structural analysis strongly suggest that BCV’s mechanism of action is inhibition of BALF5 DNA polymerase functions.

Originally developed as a treatment for smallpox, BCV has since demonstrated potent antiviral activity against orthopoxviruses, polyomaviruses, papillomaviruses, herpesviruses, and adenoviruses in both cell culture systems and animal models (31, 35–39). The recent clinical trial using an IV formulation of BCV to treat hematopoietic stem cell transplant recipients has demonstrated the robust clinical efficacy of BCV treatment in reducing the adenovirus load in immunocompromised patients (40). In addition, a patient with mixed dsDNA infection, including adenovirus, cytomegalovirus, EBV, and BK polyomavirus, was successfully treated with BCV, which led to the resolution of EBV viremia (41). Additionally, in a pediatric renal transplant recipient, BCV was also able to reduce BK and EBV viral load following the development of BK virus nephropathy (42). Collectively, these reports suggest that BCV may be capable of attenuating EBV viral activity in vivo, particularly in patients with MS.

In the present study, we evaluated the effects of BCV treatment on EBV viral reactivation in vitro using spontaneous lymphoblastoid cell lines (SLCLs) and peripheral blood mononuclear cells (PBMCs) from patients with MS and HCs. Additionally, we utilized a B lymphoblastoid cell line and PBMCs from CalHV-3–infected marmosets to determine BCV efficacy on an EBV-related herpesvirus in a translational nonhuman primate model. In both species, BCV was able to significantly inhibit gammaherpesvirus reactivation, with decreased lytic and latent viral mRNA production. These results suggest that BCV may be a promising antiviral for inhibiting EBV activity in patients with MS. Additionally, this work validates CalHV-3 infection in a marmoset as an important preclinical model for the investigation of successful EBV-targeting therapeutics. Finally, if EBV is shown in the future to play a role in MS pathogenesis, the results suggest a potential prescreening rationale for the selection of participants with MS in future clinical antiviral EBV therapeutic trials.

## Results

### Effect of BCV treatment in EBV-infected SLCLs.

**Figure 1:**
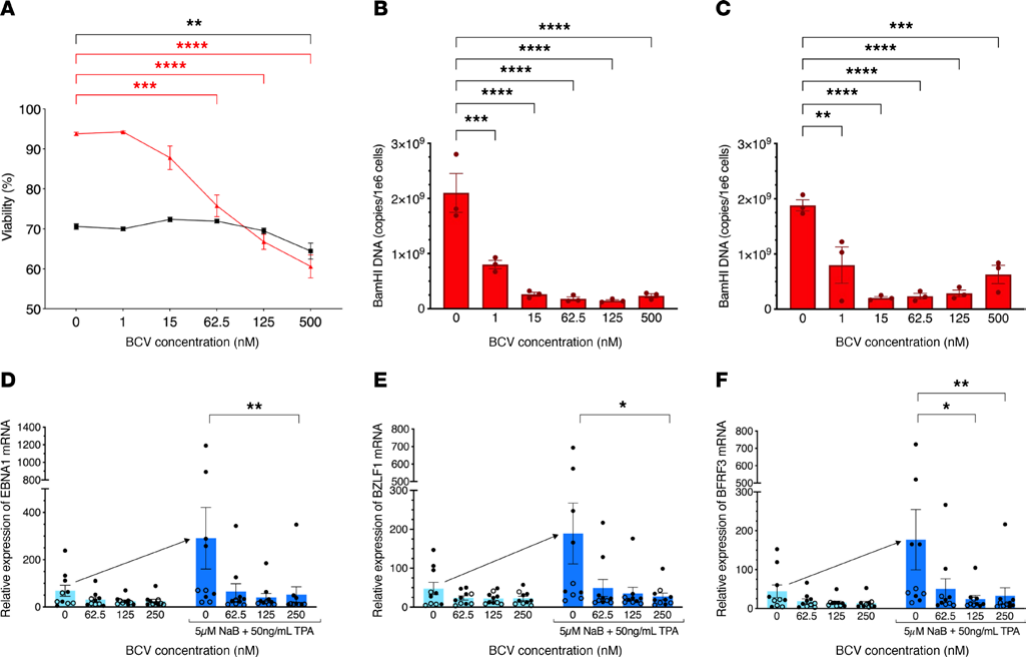
Brincidofovir decreases EBV DNA in a human EBV-infected cell line and reduces viral mRNA following reactivation. (**A**) Viability of one EBV-infected SLCL, SMS2 (red triangle), and the Ramos EBV-negative cell line (black square) after 7 days in culture with BCV. (**B** and **C**) Average EBV DNA copy number ± SEM per 1 × 10^6^ cells of the SMS2 SLCL detected by ddPCR for EBV *BamHI* DNA following 5 days in culture (**B**) and 7 days in culture (**C**) with BCV. (**D**–**F**) Average of EBV mRNA expression ± SEM in HC (open circles, *n* = 4) and MS (black circles, *n* = 6) SLCLs cultured with BCV and with (dark blue bars, to right of arrow) or without (light blue bars, under arrow) NaB+TPA to induce viral reactivation (arrow). (**D**) *EBNA1* expression; (**E**) *BZLF1* expression; (**F**) *BFRF3* expression. Data analyzed using 1-way ANOVA with Tukey’s multiple comparisons (**A**–**C**) and the Kruskal-Wallis test with Dunn’s test for pairwise comparisons (**D**–**F**), *P ≤ 0.05, **P ≤ 0.01, ***P ≤ 0.001, ****P ≤ 0.0001. BCV, Brincidofovir; EBV, Epstein-Barr virus; HC, healthy control; SMS, stable multiple sclerosis patient.

The effects of BCV were first tested on EBV-infected SLCLs that had been previously generated from HCs and patients with stable MS (SMS) or active MS (AMS) (Table 1). These SLCLs express high levels of the endogenous EBV from whom the B cell lines were generated (43, 44). The effects of BCV on the viability of an EBV-positive SLCL (SMS2) are shown in Figure 1A. Increasing concentrations of BCV demonstrated a dose-dependent effect of decreasing cell viability after 7 days in culture in the EBV-infected SLCL SMS2 compared with the EBV-negative Ramos cell line (Figure 1A). In addition, the effects of BCV on EBV viral DNA load in the SLCL SMS2 were also analyzed after both 5 and 7 days in culture (Figure 1, B and C respectively). The SLCL was cultured with BCV at concentrations between 1 and 500 nM for 7 days, and EBV DNA copy number for each concentration was determined by droplet digital PCR (ddPCR). Samples were harvested on days 5 and 7. BCV exhibited a dose-dependent effect on EBV viral load at concentrations of BCV beginning as low as 1 nM for SMS2 SLCL on day 5 (Figure 1B) and day 7 (Figure 1C). These results demonstrate that BCV selectively targets EBV-infected cells, and the lowest, most effective doses of BCV were between 1 nM and 500 nM. Therefore, subsequent experiments utilized BCV concentrations of 500 nM or less.

It is known that treating cells latently infected with EBV with tetradecanoyl phorbol acetate (TPA) induces the expression of the EBV lytic transactivator protein Zta (*BZLF1)*, through activation of protein kinase C (PKC), to drive reactivation and genome-wide gene expression (45). Sodium butyrate (NaB) is an inhibitor of histone deacetylases and leads to a broad increase in chromatin accessibility and gene expression (46). Therefore, we used the combined treatment of TPA and NaB to induce EBV reactivation in 10 SLCLs derived from healthy controls and patients with MS (Table 1). To analyze viral gene expression, EBV transcripts for the latent EBV nuclear antigen 1 (*EBNA1*) (Figure 1D), the lytic immediate early EBV Zta viral transcription and replication factor (*BZLF1*) (Figure 1E), and the late EBV small capsid protein BFRF3 (*BFRF3*) (Figure 1F) were measured. Treatment of all 10 SLCLs with 5 μM NaB and 50 ng/mL of TPA led to an approximately 4-fold increase of all 3 EBV transcripts examined over the 7-day time course (Figure 1, D–F, arrow). The effect of BCV on the expression of reactivated EBV mRNAs resulted in a reduction of all 3 EBV mRNA transcripts by approximately 80% in all SLCLs (Figure 1, D–F). This reduction was statistically significant (Figure 1, D–F) for all mRNAs at the highest concentration of BCV (250 nM) and at the 125 nM dose of BCV for *BFRF3* (Figure 1F). Despite the stratification of samples, there was no substantial difference between healthy controls and patients with MS at any concentration of BCV-induced reduction of reactivated EBV in SLCLs (*P >.*05; *t* test). In addition, there was no notable difference in the degree of reactivation between HCs and patients with MS following treatment with NaB and TPA (*P >.*05; *t* test). These results suggest that BCV effectively reduces the relative expression of latent, immediate lytic, and late lytic EBV genes in reactivated EBV in the SLCLs.

### BCV decreases the viral load in EBV reactivated from PBMCs.

**Figure 2:**
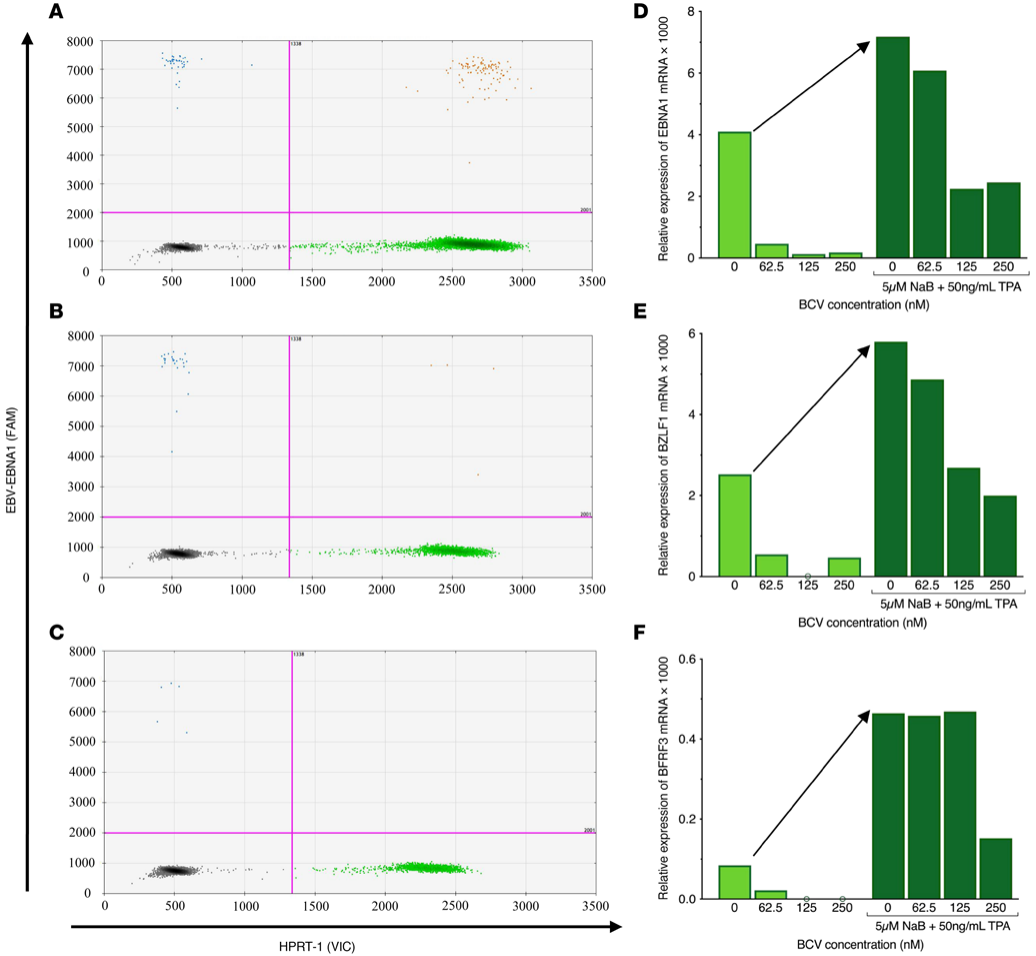
Brincidofovir reduces EBV mRNA expression in PBMCs from an individual with active EBV infection following viral reactivation. (**A**–**C**) Representative ddPCR plots of *EBNA1* detection from HC6 PBMCs cultured (**A**) without NaB+TPA and no BCV, (**B**) with NaB+TPA and no BCV, and (**C**) with NaB+TPA and 125 nM BCV. Y axis represents the FAM-MGBNFQ fluorescent amplitude, corresponding to *EBNA1* detection in this plot (blue dots + orange dots, upper quadrants). X axis represents the VIC-MGBNFQ fluorescent amplitude, which corresponds to the *HPRT* housekeeping gene (green dots, bottom right quadrant). Lower left quadrant represents droplets negative for both *EBNA1* and *HPRT* (gray dots). (**D**–**F**) Quantification of EBV mRNA expression in HC6 PBMCs cultured with BCV and with or without NaB+TPA to induce viral reactivation (arrow). Treatment with BCV and no NaB+TPA (light green bars, under arrow); treatment with BCV plus NaB+TPA (dark green bars, right of arrow). (**D**) *EBNA1* expression; (**E**) *BZLF1* expression; (**F**) *BFRF3* expression. Open circle intersecting x-axis indicates mRNA expression undetected by ddPCR. BCV, Brincidofovir; EBV, Epstein-Barr virus; HC, healthy control.

To determine if BCV is also effective at reducing reactivated EBV from PBMCs, the relative expression of reactivated EBV mRNA genes was measured in PBMCs from 23 individuals (Table 2), including HCs, patients with MS, and one healthy donor with an active EBV infection at the time of sample collection (Table 2, HC6). Although this individual was healthy, as he was recruited to be a bone marrow donor at the time of collection, he was IgM positive for an EBV infection and had a high EBV *BamHI* DNA copy number of 23,700 copies per 1 × 10^6^ cells (Table 2). In addition, EBV mRNA was detected in freshly isolated PBMCs from this individual. A representative ddPCR plot is shown in Figure 2A, in which EBV *EBNA1* mRNA is shown as positive droplets in the upper quadrants. Reactivation with NaB+TPA for 7 days generated a relative increase in EBV *EBNA1* mRNA (Figure 2B, upper quadrant drops), quantified as a 1.75 increase in Figure 2D (arrow). Similar increases in *BZLF1* and *BFRF3* mRNA expressions, of 2.32 and 5.48 respectively, were also observed (Figure 2, E and F, arrows). Importantly, BCV was shown to reduce EBV mRNAs from both nonreactivated PBMCs (Figure 2, D–F, light green bars under arrow) and in NaB+TPA reactivated EBV (Figure 2, D–F, dark green bars to right of arrow) in a dose-dependent manner. A representative ddPCR plot is shown in Figure 2C and quantified in Figure 2D. These results clearly demonstrate that BCV can reduce mRNA expression of EBV genes in an individual with an active EBV infection.

**Figure 3:**
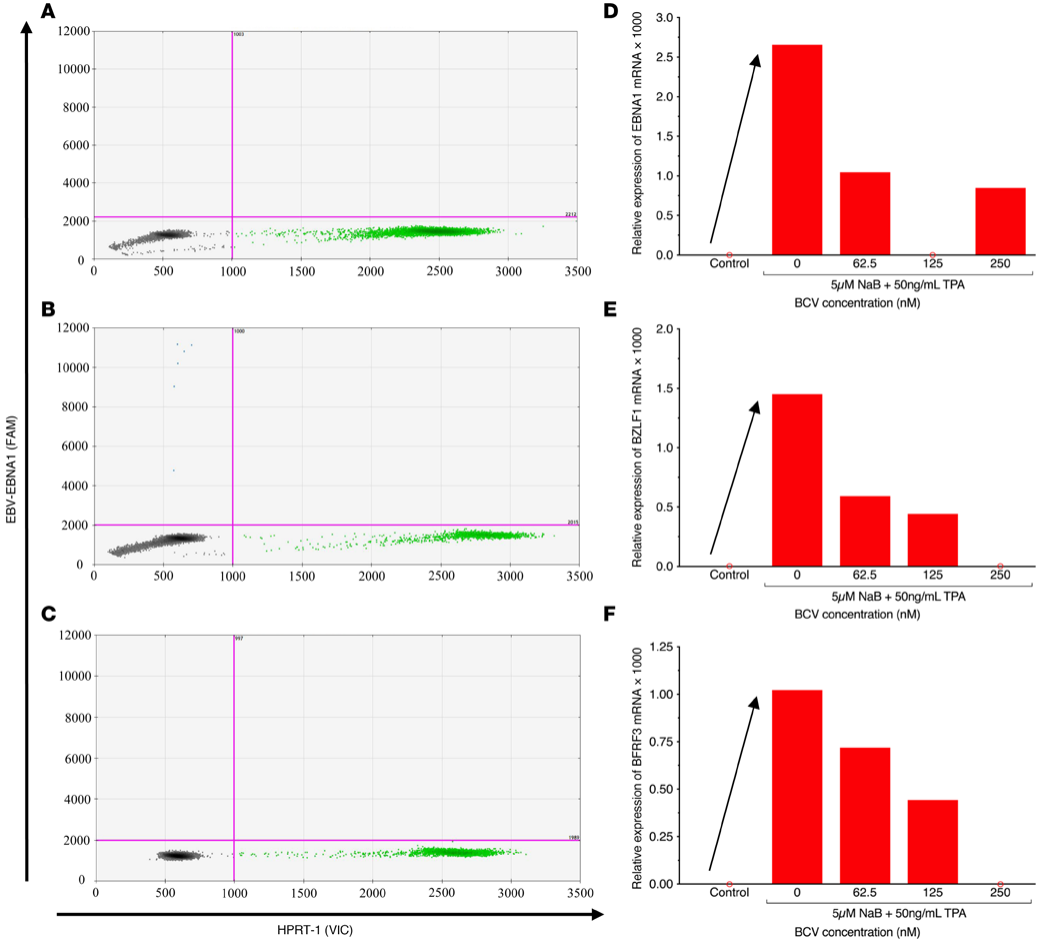
Brincidofovir reduces EBV mRNA expression in PBMCs from an individual with stable multiple sclerosis (SMS) following viral reactivation. (**A**–**C**) Representative ddPCR plots of *EBNA1* detection from SMS6 PBMCs cultured (**A**) without NaB+TPA and no BCV, (**B**) with NaB+TPA and no BCV, and (**C**) with NaB+TPA and 125nM BCV. Y axis represents the FAM-MGBNFQ fluorescent amplitude, corresponding to *EBNA1* detection in this plot (blue dots + orange dots, upper quadrants). X axis represents the VIC-MGBNFQ fluorescent amplitude, which corresponds to the *HPRT* housekeeping gene (green dots, bottom right quadrant). Lower left quadrant represents droplets negative for both *EBNA1* and *HPRT* (gray dots). (**D**–**F**) Quantification of EBV mRNA expression in SMS6 PBMCs cultured with BCV and with or without NaB+TPA to induce viral reactivation (arrow). (**D**) *EBNA1* expression; (**E**) *BZLF1* expression; (**F**) *BFRF3* expression. Open circle intersecting x-axis, mRNA expression undetected by ddPCR; BCV, Brincidofovir; EBV, Epstein-Barr virus; SMS, stable multiple sclerosis patient; Control, cells treated with only media.

**Figure 4:**
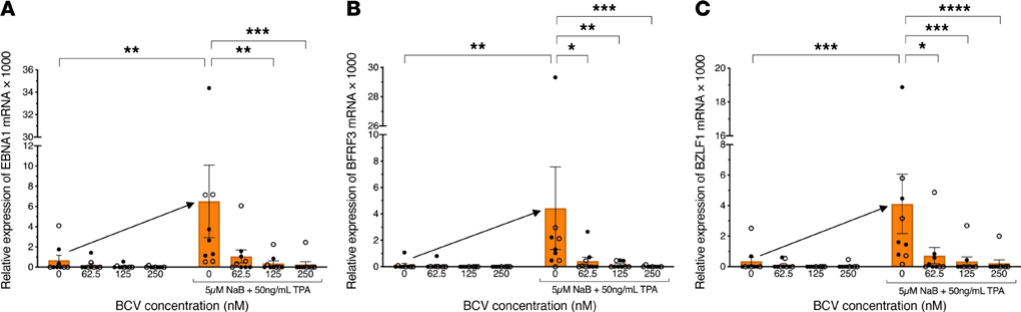
Treatment of EBV-infected human PBMCs with brincidofovir attenuates viral reactivation. (**A**–**C**) Average of EBV mRNA expression ± SEM in human PBMCs cultured with BCV and with or without NaB+TPA to induce viral reactivation (arrow). HC PBMCs, open circles (*n* = 4). MS PBMCs, black circles (*n* = 5). (**A**) *EBNA1* expression; (**B**) *BZLF1* expression; (**C**) *BFRF3* expression. Data analyzed using Kruskal-Wallis test with Dunn’s multiple comparisons, ****P ≤ 0.0001. H, healthy control; MS, multiple sclerosis patient.

Similarly, the effect of BCV on reactivated PBMCs from both healthy individuals and patients with MS not experiencing an active EBV infection was determined. A ddPCR plot is shown in Figure 3A from a representative patient with MS (Table 2, SMS6), demonstrating the typical observation of no EBV mRNA from fresh PBMCs. However, upon reactivation with NaB+TPA for 7 days, EBV *EBNA1* mRNA was detected (Figure 3B, drops upper quadrant) and quantified as a dramatic increase in EBV *EBNA1* mRNA (Figure 3D, arrow). Again, a similar increase in *BZLF1* and *BFRF3* mRNA was also observed (Figure 3, E and F respectively, arrows). Treatment of EBV-reactivated PBMCs with BCV demonstrated a reduction in the expression of EBV *EBNA1* mRNA, as shown by fewer drops (Figure 3C, upper left quadrant) and quantified in Figure 3D as a dose-dependent reduction. The relative expressions of *BZLF1* and *BFRF3* mRNAs in MS patient SMS6 displayed similar dose-dependent reductions in response to BCV as *EBNA1* mRNA (Figure 3, D–F). These representative results are supported by group analysis of 9 individuals (4 HCs and 5 patients with MS) in which there was a greater than 30-fold increase in all 3 EBV mRNA transcripts upon reactivation with NaB+TPA (Figure 4). Importantly, there was a greater than 90% reduction in all EBV-reactivated mRNAs following treatment with 125 nM and 250 nM BCV (Figure 4). No differences were observed in EBV mRNA detection pre- and postreactivation between healthy donors and MS patients (Figure 4, circles). Additionally, no marked differences were found between the magnitudes of reactivation of healthy donor PBMCs and MS patient PBMCs. Collectively, these results demonstrate that BCV can actively reduce the levels of EBV mRNAs from PBMCs of healthy individuals and patients with MS following EBV reactivation.

### BCV has a dose-dependent effect on CalHV-3 in reactivated marmoset cells.

To further explore the efficacy of BCV, we utilized another lymphocrypto-gammaherpesvirus (CalHV-3) naturally occurring in a nonhuman primate, the common marmoset (23, 25). CalHV-3 shares many phylogenetic, biological, and pathogenic similarities with EBV and is used as a translational experimental model of EBV infection and associated diseases (26, 28). A marmoset B cell line, CJ0149, endogenously expresses high levels of CalHV-3 DNA and has been shown to transcribe viral mRNA (*ORF45*, *ORF39*) in a pattern broadly consistent with lytic and latent gene expression programs of EBV homologs (*gp350*, *EBNA1*). The CalHV-3–infected CJ0149 cell line was stimulated with increasing doses of NaB+TPA to determine optimal reactivation conditions. Higher concentrations of 5 mM NaB + 20 ng/mL TPA, compared with EBV reactivation conditions from human cells, were required to reactivate CalHV-3 mRNA from the CJ0149 cell line (Supplemental Figure 1, A and B; supplemental material available online with this article; https://doi.org/10.1172/JCI195764DS1). Additionally, reactivation of CalHV-3 peaked on day 5 of treatment (Supplemental Figure 1, C–E), compared with day 7 for EBV in human cells.

**Figure 5:**
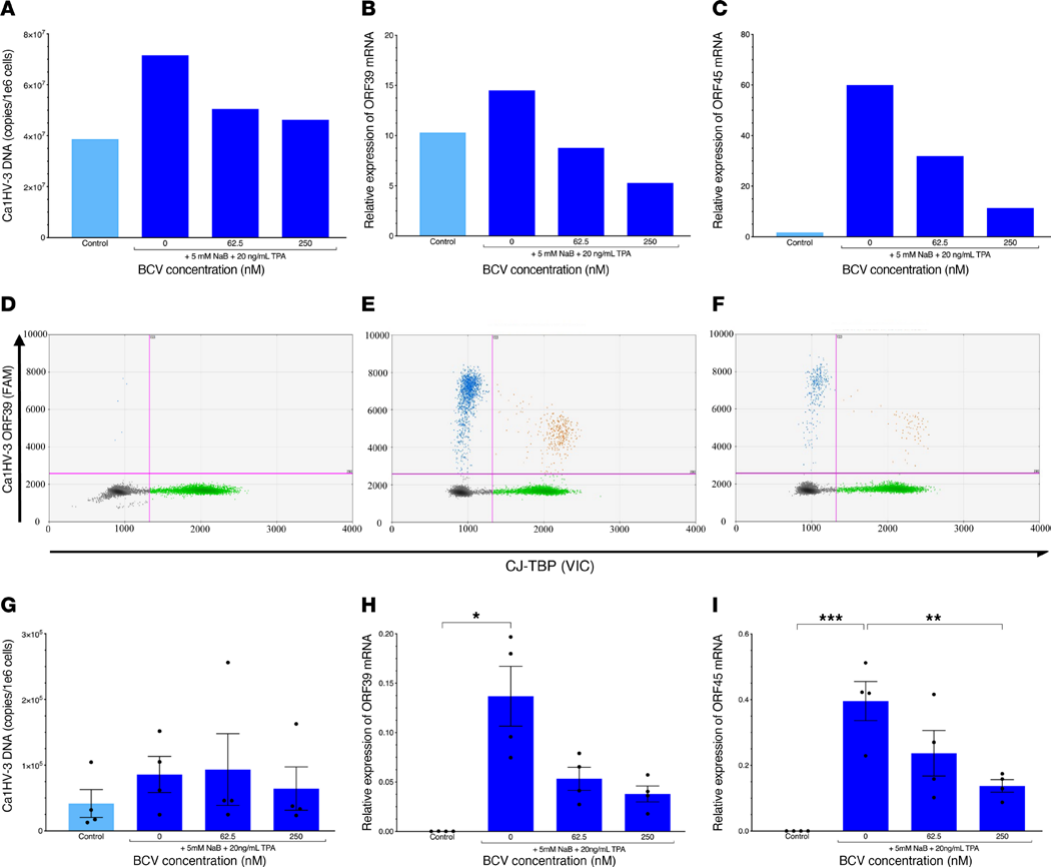
CalHV-3–infected marmoset cells treated with brincidofovir show decreases in CalHV-3 DNA and mRNA following viral reactivation assay. (**A**–**C**) Quantification of Callitrichine herpesvirus 3 (CalHV-3) DNA and mRNA expression in CalHV-3 infected cell line, CJ0149, with (dark blue bars, right) or without (light blue bar, left) NaB+TPA to induce viral reactivation. (**A**) CalHV-3 DNA expression; (**B**) *ORF39* mRNA expression; (**C**) *ORF45* mRNA expression. (**D**–**F**) Representative ddPCR plots of *ORF39* mRNA expression in marmoset #3 PBMCs cultured (**D**) without NaB+TPA and no BCV, (**E**) with NaB+TPA and no BCV, and (**F**) with NaB+TPA and 250nM BCV. Y axis represents FAM-MGBNFQ fluorescent amplitude, which corresponds to *ORF39* mRNA detection (blue dots + orange dots, upper quadrants). X axis represents the VIC-MGBNFQ fluorescent amplitude, which corresponds to the *CJ-TBP* housekeeping gene (green dots, bottom right quadrant). Lower left quadrant represents droplets negative for both *ORF39* and *CJ-TBP* (gray dots). (**G**–**I**) Mean detection ± SEM of CalHV-3 gene expression in marmoset PBMCs (*n* = 4, circles) cultured with BCV and with (dark blue bars, right) or without (light blue bar, left) NaB+TPA to induce viral reactivation. (**G**) CalHV-3 DNA expression; (**H**) *ORF39* mRNA expression; (**I**) *ORF45* mRNA expression. Data analyzed using (**G**) Kruskal-Wallis test with Dunn’s comparisons, (**H**) a Welch’s ANOVA, and (**I**) 1-way ANOVA with Tukey’s multiple comparisons, *P ≤ 0.05, **P ≤ 0.01, ***P ≤ 0.001. Control, cells treated with only media; BCV, brincidofovir.

After determining the effective reactivation conditions for the CJ0149 marmoset cell line, the effect of BCV was assessed. CalHV-3–infected CJ0149 cells were reactivated and treated with BCV (Figure 5, A–C). Reactivation with NaB+TPA demonstrated an increase in CalHV-3 DNA and CalHV-3 *ORF39* and *ORF45* mRNA, and all were inhibited by treatment with BCV in a dose-dependent manner (Figure 5, A–C). We extended these observations of the effect of BCV on CalHV-3 from the CJ0149 cell line directly to CalHV-3–infected marmoset PBMCs. As limited quantities of marmoset samples were available due to collection restrictions, conditions of PBMC reactivation with NaB+TPA were first determined. Plating 1.5 × 10^5^ marmoset PBMCs per well and incubating the cells with 5 mM NaB + 20 ng/mL TPA were the optimal conditions for reactivation during a 5-day assay (Supplemental Figure 2). As shown in the upper quadrants of Figure 5D, ddPCR of CalHV-3–positive marmoset #3 PBMCs with no reactivation showed limited expression of CalHV-3 *ORF39* mRNA, which dramatically increased upon stimulation with NaB+TPA (Figure 5E, upper quadrants). Treatment with BCV of these reactivated marmoset PBMCs resulted in a decrease of *ORF39* mRNA expression (Figure 5F, upper quadrants). These results are quantified in a group analysis of 4 animals, in which increases in both CalHV-3 DNA (Figure 5G) and, in particular, CalHV-3 mRNA expression (Figure 5, H and I), were observed. While treatment of CalHV-3 infected marmoset PBMCs with BCV resulted in a modest decrease in CalHV-3 reactivated DNA (Figure 5G), there was a 70% decrease in the *ORF39* mRNA at 250 nM of BCV (Figure 5H) and a statistically significant decrease in expression of *ORF45* (Figure 5I). These results further demonstrate that BCV has a broad effect on herpesviruses and suggest the potential for the use of marmosets infected with CalHV-3 as a nonhuman primate model for antiherpesvirus therapies.

### Reactivation of EBV from human PBMCs is correlated with the production of SLCLs and suggests a rationale for the selection of patients with MS for antiherpesvirus therapy research.

**Figure 6:**
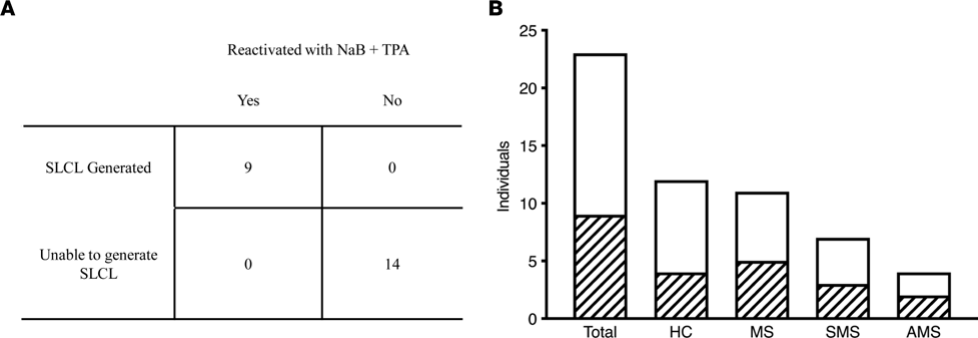
Ability to reactivate EBV in human PBMCs correlates with ability to generate a spontaneous lymphoblastoid cell line. (**A**) Table demonstrating correlation between successful reactivation of EBV and ability to generate an SLCL for 23 individuals (Fisher’s exact test, *P* < 0.0001). (**B**) Representation of number of individuals in which EBV was successfully reactivated (shaded area) compared with the total number of individuals tested. Total PBMCs reactivated: 9/23, 39%; HC PBMCs reactivated: 4/12*,* 33%*;* MS PBMCs reactivated: 5/11, 45%; SMS PBMCs reactivated: 3/7*,* 43%; AMS PBMCs reactivated: 2/4, 50%. HC, healthy control; MS, multiple sclerosis patient; SMS, stable multiple sclerosis patient; AMS, active multiple sclerosis patient; SLCL, spontaneous lymphoblastoid cell line.

A major theme of this study was that treatment with BCV inhibited the reactivation of EBV from EBV-infected cell lines (SLCLs) (Figure 1) and ex vivo PBMCs (Figure 4). The results in Table 2 demonstrate that 5 μM NaB + 50 ng/mL TPA reactivation of EBV from PBMCs was variable between individuals. Indeed, from 23 individuals tested, reactivation was observed in 9 (40%) (Table 2 and Figure 6). There were no apparent differences in reactivation between healthy control PBMCs and patients with MS, or between patients with MS in either the stable or active phase of their disease (Figure 6B). Of interest was the observation that, in those 9 individuals, either patients with MS or healthy controls, whose PBMCs were able to reactivate EBV after treatment with NaB+TPA, SLCLs were successfully generated from all 9 (Table 2 and Figure 6A). Conversely, in those 14 individuals in which EBV could not be reactivated, no SLCLs were generated (Table 2 and Figure 6A). These results suggest a significant correlation between the ability to reactivate EBV from PBMCs in response to ex vivo stimulation and the capacity to generate EBV-infected SLCLs. As treatment with an anti-EBV compound like BCV was particularly effective in the inhibition of EBV reactivated from PBMCs, consistent with its mode of action as a viral DNA polymerase inhibitor (47), these observations suggest a rationale for screening of individuals, such as patients with MS, for inclusion in an anti-EBV clinical trial.

## Discussion

Although the cause of MS is unknown, it is clear that multiple biological processes, including genetic, immunological, and environmental factors, are associated with disease pathogenesis (2). In particular, the role of EBV in MS has recently garnered renewed interest because it is considered that EBV exposure may be a necessary but insufficient trigger for this disease (2, 3, 5, 13, 48). It has been suggested that the reactivation of EBV from its latent to its lytic phase could play a role in stimulating neuroinflammation (49). Because of the potential for EBV, or other herpesviruses, to dysregulate the host immune response and trigger MS in predisposed individuals, the use of antivirals as potential therapeutic treatments for MS is of recent interest.

The focus of this study was the effect of the drug brincidofovir (BCV) on EBV and a nonhuman primate homolog of EBV known as CalHV-3, another lymphocrypto-gammaherpesvirus (23, 25, 26). BCV is a lipidconjugate of the FDA-approved cidofovir (CDV) that has broad-spectrum antiviral activity against several double-stranded deoxyribonucleic acid (dsDNA) viruses by acting as an alternative substrate inhibitor of viral DNA polymerase to inhibit viral replication (31). BCV is an FDA-approved treatment for smallpox, a double-stranded DNA virus like EBV, and is currently in a phase 2a clinical trial for the treatment of individuals with adenovirus and CMV (50). BCV is being considered for its efficacy in EBV-associated lymphoproliferative disease and could now be investigated as a potential antiviral agent in MS, in which EBV is thought to play a role (3, 18). This study aimed to determine the efficacy of BCV in preventing EBV reactivation in human cells from healthy controls and patients with MS. Additionally, the effect of BCV on CalHV-3 in the common marmoset was studied to further establish the role of CalHV-3 as a preclinical animal model of EBV. Finally, observations were made that suggest a potential prescreening tool using reactivation of EBV from PBMCs for use as an inclusion criterion in future clinical trials targeting EBV in MS.

In the present study, the effect of BCV on EBV was first explored in human SLCLs and PBMCs. The EBV-infected B cell lines employed in this study, which have been previously generated from both patients with MS and healthy controls, are unique. Unlike conventional lymphoblastoid cell lines that are uniformly transformed by exogenous infection with the B95 variant of EBV, the SLCLs have been spontaneously transformed by the endogenous EBV associated with each individual (43, 44). All SLCLs had high EBV viral DNA loads (greater than 1 × 10^9^ EBV copies per million cells) in which BCV had a dose-dependent inhibitory effect, even with these extraordinarily high viral loads (Figure 1, B and C). This is in marked contrast to the EBV DNA amount observed in fresh PBMCs, which is typically undetected or at very low viral concentrations in some patients with MS and controls (Table 2). Because EBV DNA viral loads are either low or undetected, we induced the expression of EBV gene products in PBMCs and measured EBV mRNA levels.

We reactivated EBV in both SLCLs and fresh PBMCs with tetradecanoyl phorbol acetate (TPA) and sodium butyrate (NaB) (45, 46), as measured by EBV transcripts for the latent EBV nuclear antigen 1 (*EBNA1*), the lytic immediate early EBV Zta viral transcription and replication factor (*BZLF1*), and the late EBV small capsid protein (BFRF3). A 4-fold reactivation of EBV from baseline was observed in all NaB+TPA-treated SLCLs (Figure 1), and a significantly greater (40-fold) increase in EBV viral gene expression was observed from NaB+TPA-treated PBMCs (Figure 4 and Table 2). The greater reactivation of EBV from PBMCs versus SLCLs could be a result of the much higher viral loads observed in SLCLs. This is supported by the observations from one healthy donor (HC6, Table 2) who was experiencing an active EBV infection at the time of PBMC collection and demonstrated a high viral load (23,700 copies of EBV/million cells). Reactivation of EBV from this donor resulted, on average, in a 3-fold increase in EBV mRNA expression (Figure 2).

Following the EBV reactivation conditions established for SLCLs and human PBMCs, we tested the effect of BCV on the induced expression of EBV mRNAs. BCV was highly effective at reducing the expression of both latent (*EBNA1*) and lytic (*BZLF1*, *BFRF3*) EBV mRNAs in a dose-dependent manner (Figures 1–3). Moreover, this inhibition was observed at surprisingly low concentrations in the nanomolar range. BCV inhibition of reactivated EBV was observed in long-term EBV-infected lymphoblastoid cell lines expressing high levels of EBV and in reactivated PBMCs from an individual with an active EBV infection expressing moderate levels of EBV prior to reactivation. In particular, a greater than 90% inhibition of EBV transcripts was observed in reactivated PBMCs from 10 individuals with low or no EBV viral genomes detected (Figure 4) treated with 250 nM BCV in 7 days of culture. Importantly, no differences were observed between healthy controls and patients with MS, demonstrating that the effect of BCV was not disease specific, but, instead, limited the expression of EBV mRNAs following reactivation in any EBV latently infected cell. Indeed, as EBV is latent in the vast majority of people, the role that EBV plays in the pathogenesis of MS has been suggested to be associated with the in vivo reactivation of the virus (5, 51). An antiviral therapy such as BCV that targets reactivation and is consistent with its mode of action as a viral DNA polymerase inhibitor may therefore be an excellent candidate for EBV-associated diseases, especially at lower doses such as those that are currently being tested clinically for the treatment of human viruses with BCV (40, 47). When compared with other commonly used and FDA-approved antivirals, such as ganciclovir and foscarnet, BCV consistently exhibited a lower EC_50_ (52). Furthermore, BCV showed a low-range EC_50_ of antiviral activity for HHV-6 and clinical benefits against post-HSCT HHV-6 viral load and encephalitis as well (53).

This broad effect of BCV on herpesviruses was also evaluated in a nonhuman primate model in the common marmoset, which is known to be naturally infected with another lymphocrypto-gammaherpesvirus, CalHV-3 (23, 25). CalHV-3 shares many biological similarities with EBV and can be used as a translational model of EBV infection (26, 28). Moreover, there is a marmoset lymphoblastoid cell line (CJ0149) derived from a marmoset B cell lymphoma that is persistently infected with high levels of CalHV-3 (23). Although a higher dose of NaB+TPA was required to reactivate CalHV-3 from CJ0149 cells than EBV from human cells, BCV was again effective at reducing CalHV-3 DNA and both latent (*ORF39*) and lytic (*ORF45*) mRNA gene expressions in a dose-dependent manner (Figure 5, A–C). As previously observed with EBV, BCV treatment of reactivated CalHV-3 in PBMCs of infected marmosets again demonstrated a significant decrease in herpesvirus mRNA expression (Figure 5, G–I). CalHV-3–infected marmosets are actively being considered as a translational nonhuman primate model to explore the role that herpesviruses may play in chronic diseases of humans such as multiple sclerosis and Alzheimer’s disease (54). The investigation of the efficacy of BCV on CalHV-3 infection in the common marmoset is ongoing. The utility of animal models of both herpesvirus infection and MS will provide important results to aid the development of therapeutics such as BCV (55) For example, experimental autoimmune encephalomyelitis (EAE) in the nonhuman primate may provide a better translational model of therapeutic testing to humans than experiments using a mouse model (56). Given the results of our study, we believe that the efficacy of BCV or any antiviral in in vivo models of MS would support the hypothesis for a role of viruses in the pathogenesis of MS.

In conclusion, we have evaluated the effects of BCV on viral reactivation in vitro using human SLCLs, human PBMCs, the marmoset CJ0149 cell line, and marmoset PBMCs. BCV significantly inhibited the reactivation of both EBV and CalHV-3, as indicated by decreased lytic and latent viral mRNA production. The results in this study have implications for the clinical use of BCV (or any antiviral compound) as an antiherpesvirus treatment in MS. Since greater than 90% of all individuals are latently infected with EBV, it has been challenging in MS trial design to select a patient population that may most benefit from anti-EBV therapy (6, 57). Indeed, we have optimized conditions to ex vivo reactivate EBV from PBMCs that were effective in approximately 40% of individuals (Figure 6). Reactivation of EBV was 100% correlated with the capacity to generate SLCLs from the same individual. Failure to reactivate EBV resulted in the inability to generate SLCLs (Figure 6A). Moreover, there was no difference in the ability to reactivate EBV from patients with MS or controls or from stable or active patients with MS (Figure 6B). These observations suggest a rationale for the selection of those individuals in which EBV can be stimulated ex vivo to express EBV-related gene products and who may be sensitive to the effects of anti-viral EBV therapies such as BCV.

## Methods

### Sex as a biological variable.

Our study utilized samples from male and female healthy controls and patients with MS. For experiments using nonhuman primate cells, both male and female marmosets were studied. Our study did not make any distinction between sexes as sex was not considered as a biological variable.

### Human sample selection.

Blood samples from 23 individuals were used in the study (Table 2). AMS was defined as the presence of at least 1 cerebral enhancing lesion (CEL) at the time of blood collection; patients with SMS had no CEL at the time of sampling (43, 44). Demographic information, including age, sex, and race, were provided when available. Any unknown demographic information is noted in the relevant tables.

### PBMC extraction from whole blood.

PBMCs were extracted from whole blood using ficoll-hypaque density centrifugation. PBMCs were cryopreserved at cell densities between 1 × 10^7^ and 2 × 10^7^ cells per 1 mL.

### Generation of SLCLs.

(SLCLs were generated following procedures outlined in Monaco et al. (43). Briefly, PBMCs from healthy controls and patients with MS were thawed in RPMI-1640 medium (Gibco) supplemented with 10% FBS streptomycin (Corning). PBMCs were plated in 96-well round-bottom plates at a concentration of 1 × 10^6^ cells in 200 μL of growth medium. After 3 weeks, 2 μg/mL of cyclosporin A (Sigma Aldrich) was added to the culture medium to curtail T cell surveillance of EBV-infected B cells. Clusters of B lymphoblastoid cells were observed 3 to 6 weeks following cyclosporin A treatment and were expanded and maintained in T-25 flasks.

In the present study, 10 previously generated SLCLs were used (Table 1). Four were derived from HCs, 3 from patients with SMS patients, and 3 from patients with AMS. SLCLs were maintained in T-25 flasks for the study duration and kept at concentrations of 1 × 10^6^ cells/mL.

### Cell culture of human PBMCs and SLCLs.

The Ramos (RA 1) cell line was purchased from ATCC and stored in liquid nitrogen until use (CRL-1596). Cryopreserved cells in liquid nitrogen were thawed and counted. Cells were brought to a concentration of 1.5 × 10^6^ cells/mL in RPMI-1640 medium (Gibco), supplemented with 10% FBS (Gibco), 2 mM L-glutamine (Corning), and 100 mg/mL streptomycin (Corning). Cells were rested in an incubator at 37^o^C, 5% CO_2_ overnight. The next day, cells were recounted and brought to concentrations of 3 × 10^5^ cells/100 μL or 1 × 10^4^ cells/100 μL. 100 μL of cells were plated in a 96-well round bottom plate.

Cells were assigned to 1 of 4 experimental groups: control, BCV only, reactivated, or reactivated + BCV. Control cells received no BCV or reactivation treatment. Brincidofovir (obtained from SymBio Pharmaceuticals) was prepared at a concentration of 10 mM and kept at –80˚C for long-term storage. Dilutions were prepared using the supplemented RPMI-1640 medium and stored at –20˚C or used immediately at time of the experiment. Reactivated cells were incubated with 5 μM sodium butyrate (NaB) (Millipore Sigma) and 50 ng/mL tetradecanoyl phorbol-13- acetate (TPA) (Millipore Sigma) (58). BCV-treated cells were incubated with BCV at concentrations of 1 nM, 15 nM, 62.5 nM, 125 nM, 250 nM, or 500 nM. Cells were incubated at 37°C, 5% CO_2_ for 7 days. On day 7, cells were counted and assessed for viability using the Guava Muse Cell Analyzer (Cytek). Cells were harvested and immediately prepared for DNA and RNA extraction.

### Animal housing and demographics.

Animals were housed at AAALAC-accredited facilities in accordance with USDA regulations and the Guide for the Care and Use of Laboratory Animals by the United States National Research Council. Demographic information for animals, including sex and age in months at the time of sample collection, was obtained from colony records (Supplemental Table 1).

### Animal samples.

For sample collection procedures, animals were sedated using isoflurane inhalation via facemask. Whole blood was collected, and PBMCs were isolated using lymphocyte separation medium (LSM) (Corning). PBMCs were collected and washed with PBS. PBMCs were pelleted and either stored at –20°C for DNA extraction, resuspended in media and DNA/RNA Shield (Zymo Research) for RNA extraction, or immediately plated for cell culture purposes.

### Cell culture and chemical treatment of marmoset samples.

Marmoset PBMCs and the marmoset B lymphoblastoid cell line infected with CalHV-3 (CJ0149, a previous gift from Fred Wang of Harvard Medical School [Boston, Massachusetts, USA]) were cultured in supplemented RPMI-1640 media. Reactivated CJ0149 cells were plated at 1.5 × 10^5^ cells per well and treated with the combination of TPA and NaB at the following concentrations: 1 mM NaB + 20 ng/mL TPA, and 5 mM NaB + 20 ng/mL TPA (Supplemental Figures 1 and 2). Control cells were plated only with media. To determine the effects of BCV, CJ0149 cells and marmoset PBMCs were treated with 62.5 nM or 250 nM BCV. CJ0149 cells and marmoset PBMCs were harvested for DNA and RNA extraction after 5 days in culture.

### DNA extraction.

DNA from human samples was extracted using the MinElute Virus Spin Kit (Qiagen) according to the manufacturer’s instructions and was eluted in 50 μL elution buffer. DNA from marmoset samples was extracted using the Qiagen Blood and Tissue Kit according to the manufacturer’s instruction and was eluted in 50 μL elution buffer. A NanoDrop 2000 Spectrophotometer (ThermoFisher) was used to determine DNA concentration (ng/μL) and purity (260/280). DNA samples were stored at –20 °C.

### RNA extraction.

RNA from human and marmoset samples was extracted using the Quick-RNA Viral Kit (Zymo Research) according to the manufacturer’s instructions. Samples were treated with DNase I (Zymo Research) for 15 minutes at room temperature according to the manufacturer’s instructions. All samples were eluted in volumes between 15 μL and 30 μL of DNase/RNase-free water. A NanoDrop 2000 Spectrophotometer (ThermoFisher) was used to determine viral RNA concentration (ng/μL) and purity (260/280). RNA was either stored at –80 °C or used immediately for cDNA synthesis.

### cDNA synthesis.

Extracted RNA from humans and marmosets was diluted to a concentration of 5 ng/μL using PCR-quality water (Quality Biologicals). Next, diluted RNA was converted to cDNA using the ThermoFisher High-Capacity cDNA Reverse Transcription Kit (ThermoFisher). Samples were then placed on a GeneAmp PCR System 9700 (Applied Biosystems) and incubated according to the following protocol: 25°C/10 min, 37°C/2 hr, 85°C/5 min, 4°C/hold. Prepared cDNA was stored at 4°C for up to a week, –20°C long term, or immediately used for ddPCR.

### ddPCR.

ddPCR was performed on DNA and cDNA samples to determine levels of EBV or CalHV-3 genes of interest. Human DNA samples were first diluted to 5 ng/μL then digested with HindIII. CJ0149 DNA samples were diluted 1:200 in PCR-quality water before digestion with HindIII (Quality Biologicals). Marmoset PBMC DNA samples were not diluted prior to digestion with HindIII. Each DNA and cDNA sample was mixed with primers to amplify target gene sequences, probes for fluorescent labeling, and a ddPCR 2 × Supermix (no dUTP) (BioRad). Target gene probes (*BamHI, EBNA1, BFRF3, BZLF1, gp07, ORF39, ORF45)* were FAM-MGBNFQ labeled while housekeeping gene probes (*RPP30, HPRT, ACTB, CJ-TBP*) were VIC-MGBNFQ labeled. DNA or cDNA samples were combined with primers and probes specific to the genes of interest to a final concentration of 900 nM per primer and 250 nM per probe. Samples were added to cartridge wells in duplicate, and cartridges were placed in QX200 Droplet Generator (BioRad) to generate droplets. Droplet samples were then plated in a 96-well semi-skirted green PCR Plate (ThermoFisher). Plates were then placed on a GeneAmp PCR System 9700 Thermocycler (Applied Biosystems) and incubated with the following protocol to induce PCR amplification of target genes: 95°C/10 min, 94°C/30 sec + 59°C/1 min × 40 cycles, 98°C/10 min, 12°C/hold.

Following completion of the PCR amplification cycle, gene expression was analyzed using the BioRad QX200 Droplet Reader (BioRad) and QuantaSoft software version 1.7.4.0917 (BioRad). Intensity of fluorescence was used to determine droplet positivity according to a threshold set manually. Wells were run in duplicate, representing approximately 20,000 droplets, and the average number of positive droplets was used to calculate gene expression. DNA copies were quantified by dividing positive droplets for the gene of interest by positive droplets for the housekeeping gene. Housekeeping gene–positive droplets were divided by 2 for the presence of 2 copies of each housekeeping gene per cell. Relative expression of the RNA gene of interest was quantified by dividing the droplets positive for the gene of interest by the droplets positive for the housekeeping gene. Sequences of primers and probes for human samples are presented in Supplemental Table 2. Sequences of primers and probes utilized for marmoset samples are shown in Supplemental Table 3. All primers and probes were obtained from ThermoFisher.

### Statistics.

Data were analyzed using GraphPad Prism Version 10.1.0 (GraphPad Software) and Microsoft Excel (Microsoft). A 2-way ANOVA was used to assess whether there was an interaction between diagnosis and BCV concentration on viability. As a significant interaction between both factors was observed, subsequent 1-way ANOVAs with Tukey’s multiple comparisons were performed within both diagnosis groups. One-way ANOVAs, used in conjunction with Tukey’s multiple comparison tests, were also used to assess the differences between the mean EBV DNA amounts in an EBV-positive SLCL following treatment with BCV and to assess the effect of BCV on the CalHV-3 mRNA product, *ORF45*, in reactivated marmoset PBMCs. The remaining data did not fit the parametric assumptions of a 1-way ANOVA, and therefore, alternative tests were used. A Welch’s ANOVA was used to assess the effect of BCV on the CalHV-3 *ORF39* mRNA in reactivated marmoset PBMCs. In the reactivation and BCV treatment of CalHV-3 DNA and all EBV mRNAs from both SLCLs and PBMCs, a Kruskal-Wallis test was conducted. A Dunn’s correction for multiple comparisons was performed with the Kruskal-Wallis tests. Two-tailed *t* tests were used to evaluate the difference between HC- and MS-derived SLCLs and PBMCs following reactivation and treatment with BCV. A Fisher’s exact test was used to determine the association between the ability to reactivate PBMCs using NaB+TPA and the generation of SLCLs from the same samples. Error bars represent standard error of the mean (SEM). The specific analyses used for each experiment are included in the relevant figure legend. Statistical significance was defined as *P* <.05.

### Study approval.

HC individuals (*n* = 12) were voluntary blood donors as part of the Transfusion Medicine Blood Bank at the National Institutes of Health (Bethesda, Maryland, USA). All patients with MS were enrolled in an IRB-approved natural history study (NIH Protocol# 89-N-0045). Informed consent was obtained from all participants, or from surrogate decision makers, prior to study participation. All work involving marmosets was included in active protocols reviewed and approved by the National Institute of Neurological Disorders and Stroke (NINDS) Animal Care and Use Committees (ACUC).

### Data availability.

All data are available in the main text or the supplemental materials. Supporting data values can be accessed in the Supporting Data Values file. Additional inquiries can be addressed to the corresponding author.

## Author contributions

MCM, MH, FY, LTK, SLP, and SJ conceptualized the study. MCM, AD, MRD, EHS, SLP, XL, LTK, and SJ analyzed and interpreted the data. MH, FY, LTK, and SJ acquired funds. MCM, SLP, AD, MRD, PZ, AL, and EHS conducted experiments. MCM, SLP, EHS, AD, IB, XL, LTK, and SJ developed methodologies and contributed to study design. MH, FY, XL, LTK, IC, SJ, HN, and WF provided resources. AD, MRD, MCM, EHS, SLP, and SJ wrote the manuscript. All authors reviewed, revised, and approved the manuscript.

## Funding support

This work is the result of NIH funding, in whole or in part, and is subject to the NIH Public Access Policy. Through acceptance of this federal funding, the NIH has been given a right to make the work publicly available in PubMed Central.

- The Intramural Research Program of the National Institutes of Health (NIH).
- Cooperative and Development Research Agreements (CRADA) with SymBio Pharmaceuticals Limited, Tokyo, Japan.

## Supplementary Material

Supplemental data

Supporting data values

## Figures and Tables

**Table 1:**
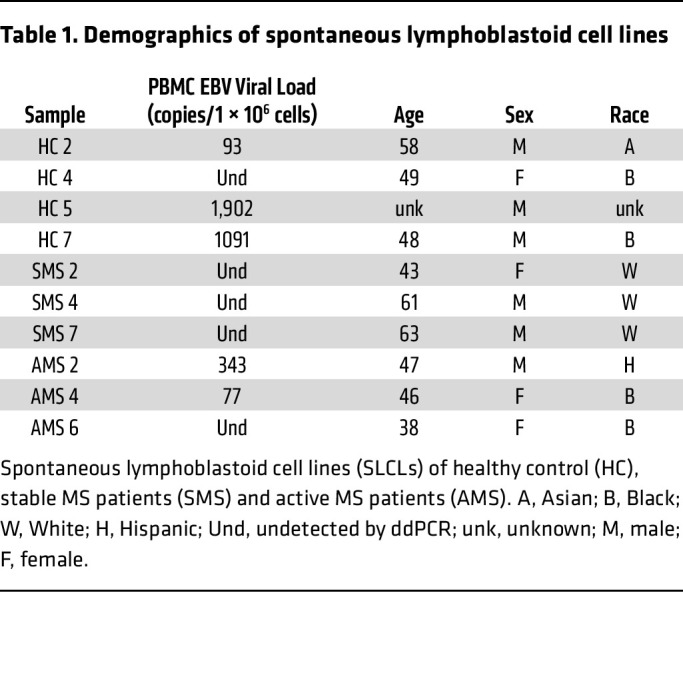
Demographics of spontaneous lymphoblastoid cell lines

**Table 2:**
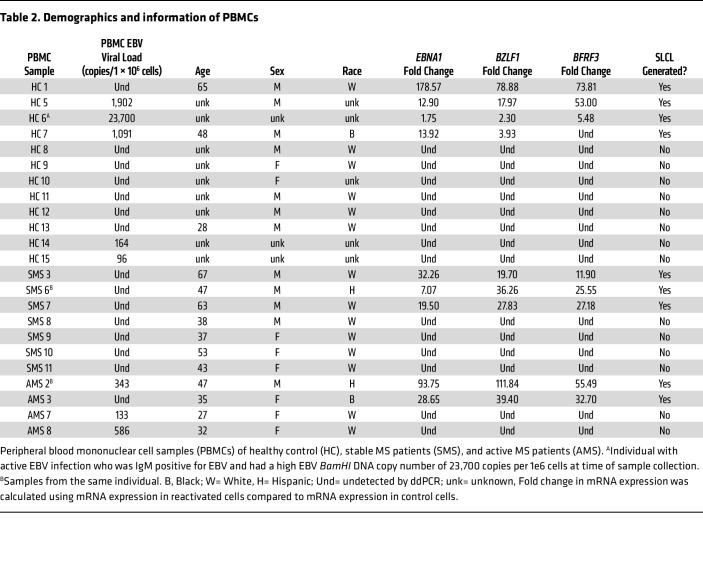
Demographics and information of PBMCs

## Version 1 11/25/2025

In-Press Preview

## Version 2 01/16/2026

Electronic publication
