# Supplementary material for: Broad-spectrum antiviral brincidofovir inhibits Epstein-Barr virus and related gammaherpesvirus in human and nonhuman primate cells
Source: J Clin Invest. 2025 Nov 25;136(2):e195764. doi: 10.1172/JCI195764 (PMC12807471; doi:10.1172/JCI195764)
Supplement: Supplemental data [file jci-136-195764-s220.pdf]

Supplemental Materials for

Broad-spectrum antiviral brincidofovir inhibits Epstein-Barr virus and related gammaherpesvirus in human and nonhuman primate cells

Abaigeal Donaldson *et al.*

Corresponding author: Steven Jacobson, jacobsons@ninds.nih.gov

This PDF file includes:

Supplemental Figures 1 and 2
Supplemental Tables 1-3

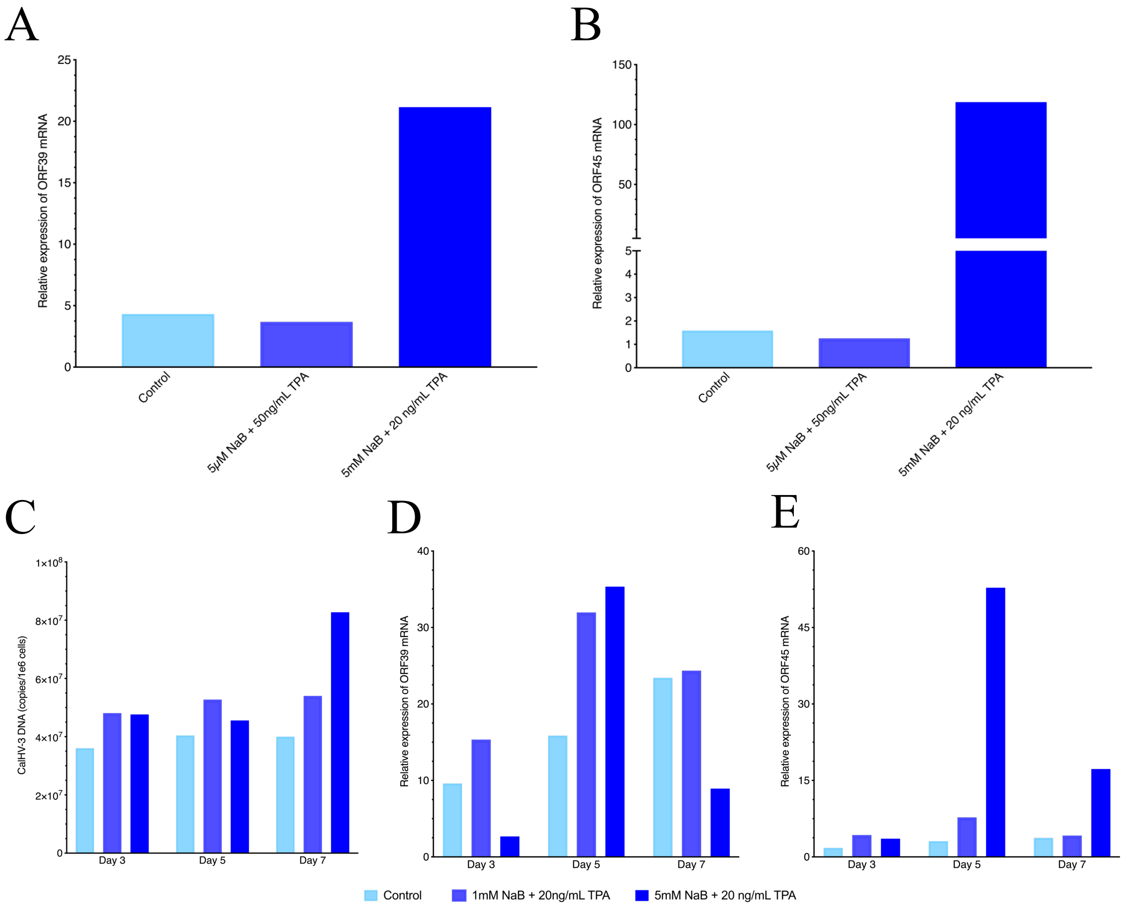

Supplemental Figure 1. Reactivation of Callitrichine Herpesvirus 3 (CalHV-3) infected marmoset CJ0149 cell line with NaB+TPA. (**A-B**) Quantification of CalHV-3 mRNAs in the marmoset B cell line CJ0149 following culture with various concentrations of NaB+TPA.(**A**) *ORF39* mRNA expression; (**B**) *ORF45* mRNA expression. (**C-E**) Quantification of CalHV-3 DNA and mRNA in CJ0149 cells following 3, 5, and 7 days of culture with various concentrations of NaB+TPA. (**C**) CalHV-3 DNA; (**D**) *ORF39* mRNA; (**E**) *ORF45* mRNA. Light blue= no NaB+TPA, medium blue= 1mM NaB + 20ng/mL TPA, dark blue= 5mM NaB + 20ng/mL TPA. Control= cells treated with only media.

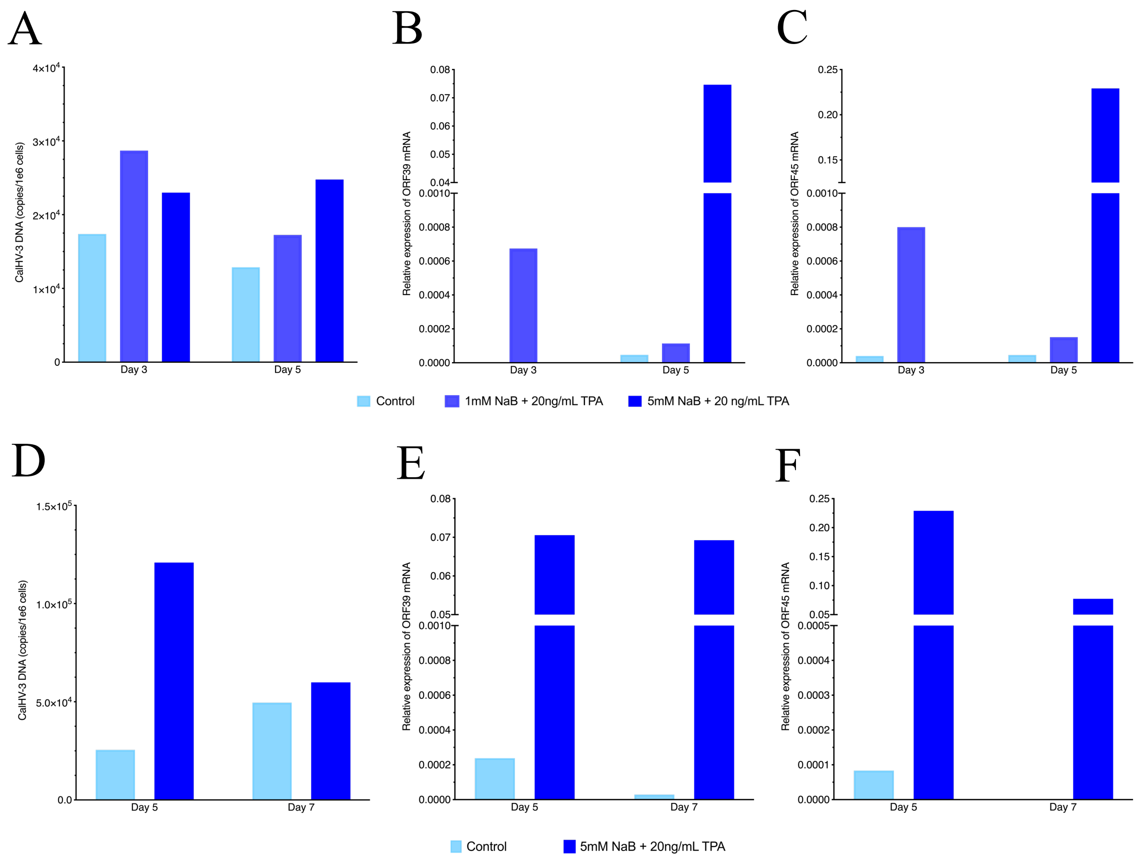

Supplemental Figure 2. Reactivation of CalHV-3 infected marmoset PBMCs with NaB+TPA. (**A-C**) Quantification of CalHV-3 DNA and mRNAs in Marmoset #1 PBMCs following culture with NaB+TPA for 3 and 5 days of culture. (**A**) CalHV-3 DNA; (**B**) *ORF39* mRNA expression; (**C**) ORF45 mRNA expression. (**D-F**) Quantification of CalHV-3 DNA and mRNAs in Marmoset #2 PBMCs following 5 and 7 days of culture with NaB+TPA. (**D**) CalHV3 DNA; (**E**) *ORF39* mRNA; (**F**) *ORF45* mRNA. Light blue= no NaB+TPA, medium blue= 1mM NaB + 20ng/mL TPA, dark blue= 5mM NaB + 20ng/mL TPA. Control= cells treated with only media.

Marmoset	Sex	Age (months)	Average Viral Load (copies per $1e^6$ cells)
Marmoset #1	F	68	25,000
Marmoset #2	M	77	15,000
Marmoset #3	F	26	60,000
Marmoset #4	M	48	15,000

Supplemental Table 1. Demographics of marmosets used in PBMC reactivation. Demographics of marmosets from which PBMC samples were used in reactivation experiments. F= female, M= male. Age is reported in months at the time of experiment. Average viral load is reported in copies per 1e6 cells and is representative of the viral load of the respective marmoset at the time of blood draw.

Gene	Sequence	Application
EBV *BamHI* fwd primer	CTTCTCAGTCCAGCGCGTTT	DNA
EBV *BamHI* rvs primer	CAGTGGTCCCCCTCCCTAGA	DNA
EBV *BamHI* probe	CGTAAGCCAGACAGCAGCCAATTGTCAG	DNA
RPP30 fwd primer	GATTTGGACCTGCGAGCG	DNA
RPP30 rvs primer	GCGGCTGTCTCCACAAGT	DNA
RPP30 probe	CTGACCTGAAGGCTCT	DNA
EBV *EBNA1* fwd primer	CATCATCATCCGGGTCTC	cDNA
EBV *EBNA1* rvs primer	CCTACAGGGTGGAAAAATGG	cDNA
EBV *EBNA1* probe	CAGGCCCCCTCCAGGTAGA	cDNA
EBV *BZLF1* fwd primer	GCAGCCACCTCACGGTAGT	cDNA
EBV *BZLF1* rvs primer	AATCGGGTGGCTTCCAGAA	cDNA
EBV *BZLF1* probe	CAGTTGCTTAAACTTGGCCCGGCA	cDNA
EBV *BFRF3* fwd primer	AACCAGAATAATCTCCCCAATG	cDNA
EBV *BFRF3* rvs primer	CGAGGCACCCCAAAAGTC	cDNA
EBV *BFRF3* probe	TTTCGGGAGGCTCAAAGA	cDNA
HPRT fwd primer*	Human HPRT1 Endogenous Control cat#4326321E (ThermoFisher)	cDNA
HPRT rvs primer*	Human HPRT1 Endogenous Control cat#4326321E (ThermoFisher)	cDNA
HPRT probe*	Human HPRT1 Endogenous Control cat#4326321E (ThermoFisher)	cDNA

Supplemental Table 2. Human EBV Sequences for ddPCR.

*Sequence is proprietary to ThermoFisher, catalogue number is provided for product reference

Gene	Sequence	Application
CalHV-3 *gp07* fwd primer	AAACCGGGTTCCCATATCAG	DNA
CalHV-3 *gp07* rvs primer	TGCCACAAACACGTATGGAA	DNA
CalHV-3 *gp07* probe	AAGCACGCAGATGCCCCA	DNA
ACTB fwd primer	AGGCGCACAGTAGGTCTGAA	DNA
ACTB rvs primer	GCGTACAAGGAAAGCACAGC	DNA
ACTB probe	CCCCATCCCAAGACCCCA	DNA
CalHV-3 *ORF45* fwd primer	GCCAATCTGTACCAAGCAATG	cDNA
CalHV-3 *ORF45* rvs primer	TTCCCTCTGTGAAATGTCCTG	cDNA
CalHV-3 *ORF45* probe	TCGGGATGCTGTGACATGTGAGATTG	cDNA
CalHV-3 *ORF39* fwd primer	ACTAAACCGCAGACATTCTCC	cDNA
CalHV-3 *ORF39* rvs primer	AAAGAGGGCTGATGCGTAC	cDNA
CalHV-3 *ORF39* probe	ACTGTGCCTTCTACGCCAAAATAGTG AT	cDNA
CJ-TBP fwd primer	CCATGACTCCCGGAATCCCTA T	cDNA
CJ-TBP rvs primer	ATAGGCTGTGGGGTCAGTCCA	cDNA
CJ-TBP probe	CAATGATGCCTTATGGCA	cDNA

Supplemental Table 3. Marmoset CalHV-3 Sequences for ddPCR.
